# Low-cost vacuum therapy as a treatment for post-operative esophago-pleural fistula after upper pole gastrectomy

**DOI:** 10.1055/a-2740-3331

**Published:** 2025-11-26

**Authors:** Jonathan Levy, Michel Queralto, Damien Marie, David Karsenti

**Affiliations:** 1 158464 Digestive Endoscopy Unit, Ramsay Santé Clinique des Cèdres, Cornebarrieu, France; 2 158464 Intensive Care Unit, Ramsay Santé Clinique des Cèdres, Cornebarrieu, France; 3 Digestive Endoscopy Unit, Pôle Digestif Paris-Bercy, Clinique Paris-Bercy, Charenton-le-Pont, France

Surgical treatment, such as upper pole gastrectomy (UPG), can be proposed to patients with gastric cardia adenocarcinoma. When mini-invasive hybrid oesophagectomy (laparoscopic gastric mobilisation with right open thoracotomy) is performed, major complications can occur in 50% of cases and pulmonary complications in 24% 1 . Post-surgical oesophago-pleural (OPF) or oeso-bronchic fistula (OBF) are some of the known complications and represent a difficult situation for clinicians. The negative pleural pressure on each inspiration prevents the fistula from closing easily. However, a second surgical intervention should be avoided. Vacuum (VAC) therapy can assure continuous drainage and favor tissue granulation. It has been shown to be effective in esophageal fistula 2 , but the literature on OPF or OBF is scarce, and there is a lack of data. Stents combined with vacuum therapy have been developed, which cover the fistula, but these devices are expensive and the French Healthcare system does not support their use. We report the case of low-cost oesophageal VAC therapy for post-surgical OBF after UPG resulting in closure of the leak.

The patient in this report was treated with VAC foam stitched to the far end of a double-lumen nasogastric tube after passage through the nasal cavity and exiting through the mouth. The tube was then pushed into the oesophagus, and the foam at the end was positioned in front of the fistula under endoscopic control, with the help of a foreign body forceps. Vacuum was applied at the end of the nasogastric tube, with either continuous or intermittent negative pressure ranging from 50–125 mmHg. The endoscopic procedure was repeated every 5 days at least four times. When the endoscopic characteristics were satisfactory, a 12 cm long fully covered self-expandable metal stent (SEMS) was inserted for 1 month. The stent was then removed. Imaging was then performed to ensure healing of the fistula.

A 71-year-old patient underwent UPG for cardia adenocarcinoma. The CT scan performed on D2 revealed a fistula (purulent fluid coming from the thoracic drain). Gastroscopy was carried out on D11. This revealed bilateral failure of the anastomotic suture with pleural fistula. A 12 cm long fully covered esophageal SEMS was inserted. The CT scan performed on D18 did not reveal any OPF. Bronchoscopy carried out on D39 revealed an OBF; the SEMS was seen from the main left bronchus. Gastroscopy carried out on D42 confirmed the presence of a bifocal fistula. A low-cost oesophageal VAC therapy, consisting of placing the foam on the fistula, was positioned in front of the anastomosis and changed every 5 days for 25 days. Another fully covered 12 cm long esophageal SEMS was then inserted. One month later, the stent was removed during gastroscopy. Imaging did not reveal any fistula or stenosis ( Video 1 ).

The video demonstrates that a low-cost vacuum therapy can lead to healing for oesophago-pleural or bronchia fistulas. This technique would be of great interest for widespread use.Video 1

Vacuum therapy has already been used for treating fistula. Low-cost oesophageal VAC therapy, consisting of placing foam in front of the fistula and used in this case and described in this report, is an inexpensive technique that can support healing of post-operative OPFs or OBFs. The patient was followed prospectively. The VAC foam was changed every 5 days for 15–25 days, with healing. No adverse effect was described. It seems primordial to treat the fistula early with vacuum therapy, an SEMS can help for healing for the first few days. This inexpensive technique should be further evaluated to confirm the promising results.

Endoscopy_UCTN_Code_TTT_1AO_2AI

## Notes

Endoscopy E-Videos https://eref.thieme.de/e-videos
*E-Videos* is an open access online section of the journal *Endoscopy* , reporting on interesting cases and new techniques in gastroenterological endoscopy. All papers include a high-quality video and are published with a Creative Commons CC-BY license. Endoscopy E-Videos qualify for HINARI discounts and waivers and eligibility is automatically checked during the submission process. We grant 100% waivers to articles whose corresponding authors are based in Group A countries and 50% waivers to those who are based in Group B countries as classified by Research4Life (see: https://www.research4life.org/access/eligibility/ ). This section has its own submission website at https://mc.manuscriptcentral.com/e-videos .
